# Residual alterations of cardiac and endothelial function in patients who recovered from Takotsubo cardiomyopathy

**DOI:** 10.1002/clc.23604

**Published:** 2021-05-06

**Authors:** Liaz Zilberman, Adi Zalik, Irina Fugenfirov, Sara Shimoni, Jacob George, Sorel Goland

**Affiliations:** ^1^ The Heart Institute, Kaplan Medical Center, Rehovot Hebrew University and Hadassah Medical School Jerusalem Israel

**Keywords:** endothelial dysfunction, longitudinal global strain, Takotusubo cardiomyopathy, tissue Doppler imaging

## Abstract

**Introduction:**

Takotsubo cardiomyopathy (TCM) is characterized by transient left ventricle dysfunction.

**Hypothesis:**

A residual cardiac and endothelial dysfunction is present in patients who recovered from TCM.

**Methods:**

In this single‐center prospective study, patients with prior TCM were included and followed for 6.4 ± 1.6 years. All underwent comprehensive cardiac function assessment, including tissue Doppler imaging (TDI) and 2‐dimensional strain (2DS) echocardiography at their first visit. The number of circulating endothelial progenitor cells and levels of proangiogenic vascular endothelial growth factor (VEGF) and its receptor (VEGF‐R) were measured. All measurements were compared with healthy controls.

**Results:**

Forty‐two women (age 58. ±8.6 years, LVEF 58.1 ± 6.1%) comprised the TCM group. Patients post‐TCM had significantly lower early velocities E′ (6 (5.0–8.0) vs. 9 (7.0–11.0) cm/s, p = .001) by TDI and higher E/E′ ratio (p = .002), lower LV global average longitudinal strain (LGS) (−18.9 ± 3.5% vs. −21.7 ± 2.3%, p = .002) and RV LGS (−20.1 ± 3.9% vs. −23.4 ± 2.8%, p = .003) were evident. There was a trend toward a higher VEGF‐R (p = .09) along with decreased VEGF/VEGF‐R ratio representing inadequate VEGF production. In‐hospital mortality was not reported and only two non‐cardiac deaths occurred at long‐term follow‐up.

**Conclusions:**

Altered TDI and 2DS indices suggest residual biventricular myocardial injury in post‐TCM patients with the apparent LV function recovery. Inappropriate production of VEGF and VEGF‐R were observed, suggesting a possible underlying endothelial dysfunction in these patients.

## 1 INTRODUCTION

Takotusbo cardiomyopathy (TCM) is an acute syndrome occurring mainly in postmenopausal women.^1^ It is induced in the vast majority of cases by emotional or physical stress. It is characterized by transient left ventricle (LV) wall motion abnormalities with no evidence of coronary artery disease.^1^, ^2^ The pathogenesis of TCM is not completely understood and several mechanisms have been proposed including catecholamine‐induced myocardial stunning, coronary spasm and microvascular dysfunction.^3^, ^4^ Recent studies have suggested that TCM is associated with endothelial dysfunction (ED) representing an important link between stress and transient myocardial dysfunction.^4^, ^5^, ^6^ Endothelial progenitor cells (EPCs) play an important role in vasculogenesis and endothelial homeostasis.^7^ Different mobilization of EPCs has been described in patients with dilated, hypertrophic and peripartum cardiomyopathy compared with healthy individuals.^8^, ^9^, ^10^, ^11^ No published data exists on endothelial function biomarkers in post TCM patients.

The incidence of TCM is underestimated due to under‐diagnosis of self‐limiting transient cardiac dysfunctions, account for up to 2% of patients with acute coronary syndrome.^12^ Data on TCM recurrence is limited.^13^ Five‐year recurrence varies from 5% to 22%^14^ and up to 20% at 10 years.^15^ InterTAK registry showed a comparable long‐term mortality risk with acute coronary syndrome patients.^16^ Pellicca et al. showed 3.5% annual rate of total mortality, and 1.0% annual rate of recurrence during a median follow‐up of 28 months.^3^

Few data have been published on residual changes in cardiac function of patients with prior recovered TCM. Only a few studies describe residual abnormalities using speckle tracking echo imaging and magnetic resonance imaging (MRI) showing systolic and diastolic deformation abnormalities that may persist despite normalization of global LV function at follow‐up of 4–20 months.^17^, ^18^, ^19^

In our study, we evaluated endothelial function measures and possible residual myocardial injury by comprehensive echocardiographic techniques in patients with apparent cardiac function recovery post‐acute TCM.

## 2 METHODS

### 2.1 Patient population

Forty‐two women with prior TCM were followed at our heart failure clinic and were enrolled at their first visit. All met the accepted diagnostic criteria for TCM according to the Mayo Clinic and the European Society of Cardiology–Heart Failure Association criteria.^14^, ^16^, ^20^ All underwent a comprehensive evaluation of the LV and RV systolic and diastolic function assessment by echocardiography, tissue Doppler imaging (TDI), and two‐dimensional speckle tracking imaging (2DS). At six‐month follow‐up, patients underwent stress echocardiography to assess LV contractile reserve. Blood samples were collected from 36 patients for circulating EPCs (CD34+, CD34+/KDR), VEGF, and VEGF‐R at time of enrollment.

All measures were compared with data from the control group that included healthy females, carefully chosen to be as close as possible in terms of age, comorbidities and BMI (Table 1).

**TABLE 1:** Demographic characteristics of patients with Takotsubo cardiomyopathy and controls at follow‐up

	TCM Patients	Controls	p value
Age, y (mean ± SD)	58.8 ± 8.6	56.3 ± 8.4	.3
Hypertension	4 (15.4%)	3 (13%)	1.0
Diabetes mellitus	—	—	
Hyperlipidemia	5 (19.2%)	3 (13%)	.7
BMI, kg/m^2^	27 ± 1	26 ± 4	1
Current smoker	3 (11.5%)	2 (8.7%)	.5
Medication			
β‐Blockers	2 (7.7%)	3 (13%)	.6
ACEI/ARB's	4 (15.4%)	3 (13%)	.7

Abbreviations: ACEI, angiotensin converting enzyme inhibitor; ARB's, angiotensin receptor blockers; BMI, body mass index; TCM, Takotsubo cardiomyopathy.

The study was approved by our institution's medical ethics review board and informed consent was obtained from all patients for being included in the study.

### 2.2 Echocardiography

Echocardiographic measurements were obtained according to the principles described in the Recommendations for Chamber Quantification.^21^ Ejection fraction measurements were based on biplane Simpson's method. Peak velocities of early (E) and late (A) diastolic filling and deceleration time were assessed by pulse‐wave Doppler.

Early (E′), late (A′) and systolic (S′) diastolic velocities were assessed by TDI. Contractile reserve by stress echocardiography (Bruce protocol) was defined as an increase in LVEF ≥5% at target heart rate. The heart rate recovery (HRR) was calculated as the difference between peak heart rate and heart rate at the first minute of recovery.

### 2.3 2DS analysis

For 2DS analysis, the LV and RV myocardium images were obtained with a frame rate > 50 Hz. 2DS and strain rate (SR) were performed by offline semiautomatic analysis. Raw data were stored digitally as DICOM cine loops and transferred for offline analysis to a workstation with the EchoPAC software (PC Dimension version 5.0.1; GE Vingmed Ultrasound, Horten, Norway).

2DS and SR were measured in the 3‐apical views (and then averaged) to determine longitudinal 2DS and SR; and in the parasternal short‐axis views at the papillary muscle level to determine circumferential and radial 2DS and SR. Myocardial rotation in the parasternal short‐axis view was measured at the mitral valve and apical levels. RV longitudinal strain was assessed from the free wall; and the base, mid and apical segments were averaged.

### 2.4 Isolation of EPC's by florescence‐activated cell sorting, VEGF and VEGF‐R ELISA

In all patients, the number of CD34+, CD34+/KDR+ cells were quantified and VEGF and VEGF‐R (sFlt1) levels were obtained in the post‐TCM women and controls.

### 2.5 Isolation of peripheral blood mononuclear cells

Human peripheral blood mononuclear cells were isolated from whole blood by a ficoll gradient. Peripheral blood mononuclear cells were taken for fluorescence‐activated cell sorting (FACS) staining, and plasma was removed and stored at −80°C for an enzyme‐linked immunosorbent assay (ELISA).

### 2.6 Determination of circulation EPCs by FACS

The number of circulating EPCs (CD34+ and CD34+/KDR) was quantified by FACS analysis. One million cells were stained by phycoerythrin anti CD34+ (Miltenyi Biotech) and allophycocyanin anti‐VEGF receptor 2 (KDR R&D System). The various EPCs phenotype assessed were CD34+ and CD34+/KDR+.^7^, ^22^

### 2.7 ELISA for VEGF and VEGF‐R

Serum levels of human VEGF and of VEGF‐R were assessed by a Quantikine ELISA kit (R&D System) according to the manufacturer's instructions and analysis protocol.

### 2.8 Statistical analysis

After evaluation of normality by Kolmogorov–Smirnov tests, continuous variables that were normally distributed are presented as mean and SD. Continuous variables that were not normally distributed are presented as median (25th–75th percentile). A two‐sided *t*‐test was performed to determine the statistical significance of differences in parameters between women with post‐TCM and controls, and a Fisher exact test was used for categorical variables. A p value <.05 was considered significant. Original data were also compared between the two groups using a nonparametric Mann–Whitney *U* test. Pearson's correlation coefficient was used for correlation analysis. Statistical analysis was performed using IBM SPSS version 21.0 (Armonk, NY).

## 3 RESULTS

Forty‐two women (mean age 58.8 ± 8.6 years) with TCM were enrolled at their first visit after their index event in the outpatient heart failure clinic at 3.5 ± 2.8 months from the acute presentation. The demographic characteristics of these patients at time of enrolment are presented in Table 1. Thirty‐six women with similar age (56.3 ± 8.4 years), comorbidities and medications comprised the control group.

### 3.1 Clinical presentation at the index event

Clinical characteristics and complications at TCM presentation are presented in Table 2. In 26 of the TCM group, provoking stressors were identified: 20 (77%) cases were associated with emotional and the rest (6, 23%) with physical triggers. All but one were admitted to the ICCU at the index event. All patients presented with typical chest pain and elevated troponin levels of 2.3 ± 1.5 ng/ml (normal <0.04 ng/ml). Sixty‐four percent had ST segment elevation on ECG, and in 14.3% QT segment prolongation was observed. Atrial fibrillation occurred in 4.8% and in one patient the clinical course was complicated with ventricular tachycardia. All underwent coronary angiography during the first hours (<8 h) after symptom occurrence. Mean LVEF at presentation was 29 ± 8%. The vast majority presented with an apical anatomical variant, but one patient (2.4%) had an inverted variant, and in three patients (7%) biventricular involvement was noted.

**TABLE 2:** Clinical presentation of patients with prior Takotusubo cardiomyopathy at the index event

	Mean ± *SD n* (%)
Age (years)	58.8 ± 8.6
Gender female	42 (100)
ECG‐ST segment elevation	27 (64)
QT segment prolongation	2 (4.8)
Troponin (ng/ml)	2.3 ± 1.5
LVEF %	29 ± 8
Anatomical variant: Apical Inverted Biventricular	38 (90) 1 (2.4) 3 (7)
Pulmonary congestion/edema	8 (19)
LV outflow tract obstruction	2 (4.8)
MR (moderate to severe/severe)	1 (2.4)
Atrial fibrillation	1 (2.4)
Ventricular tachycardia	1 (2.4)
Mechanical ventilation	1 (2.4)

Abbreviations: ECG, electrocardiogram; LV, left ventricle; LVEF, left ventricular ejection fraction; MR, mitral regurgitation.

Acute in‐hospital complications at index event are presented in Table 2.

No in‐hospital mortality cases were reported. At their first follow‐up visit all exhibited LV systolic recovery (LVEF≥55%).

### 3.2 Echocardiography

All post‐TCM patients had normal LVEF (mean 58.1 ± 6.1%) at enrollment (3.5 ± 2.8 months) and good LV contractile reserve at 6 months. LV diastolic diameter, LVEF and diastolic parameters by Doppler echo were similar in post‐TCM patients and controls (Table 3). However, compared with control subjects, post‐TCM women had a slightly larger LA area (17 (16‐21) vs. 15 (11‐17) cm^2^ p = .01), significantly lower TDI parameters such as early E′ septal (p ˂ .001), systolic S′ septal velocity (p = .004) and increase E/E′ ratio (p = .002) representing higher filling pressures.

**TABLE 3:** Comparison of echocardiographic parameters between patients with prior Takotusubo cardiomyopathy and controls

	TCM patients *n* = 42	Controls *n* = 36	p value
EF %	58.1 ± 6.1	60.1 ± 4.4	.3
LVEDd (mm)	43 (41–47)	41 (38–45)	.3
IVS (mm)	9 (7–11)	10 (9–10)	.4
PW (mm)	8.1 ± 1.5	8.7 ± 1.6	.4
LA area (cm2)	17 (16–21)	15 (11–17)	.01
PAP (mmHg)	27.5 (24–31)	22 (21–27)	.08
E (cm/sec)	70.5 (57–77)	65 (60–76)	.7
A (cm/sec)	81.4 (±16.9)	63.1 ± 15.9	.01
E′ septal (cm/sec)	6 (5.0–8.0)	9 (7.0–11.0)	.001
E/E′ septal	11 (7.9–14.3)	7.9 (5.9–9.0)	.002
S′ septal (cm/sec)	6 (6.0–7.0)	8 (7.0–9.0)	.004
E′ RV (cm/sec)	11.4 ± 3.4	12.7 ± 2.8	.1
S′ RV (cm/sec)	12.1 ± 2.5	13.0 ± 1.5	.1

Abbreviations: A, late transmitral flow velocity; E, early transmitral velocity; E′, early diastolic; S′, systolic tissue Doppler velocity; EF, ejection fraction; IVS, interventricular septum; LA, left atrium; LVEDd, left ventricular end diastolic diameter; PAP, pulmonary artery pressure; PW, posterior wall; RV, right ventricle; TCM, Takotusubo cardiomyopathy.

**FIGURE 1:**
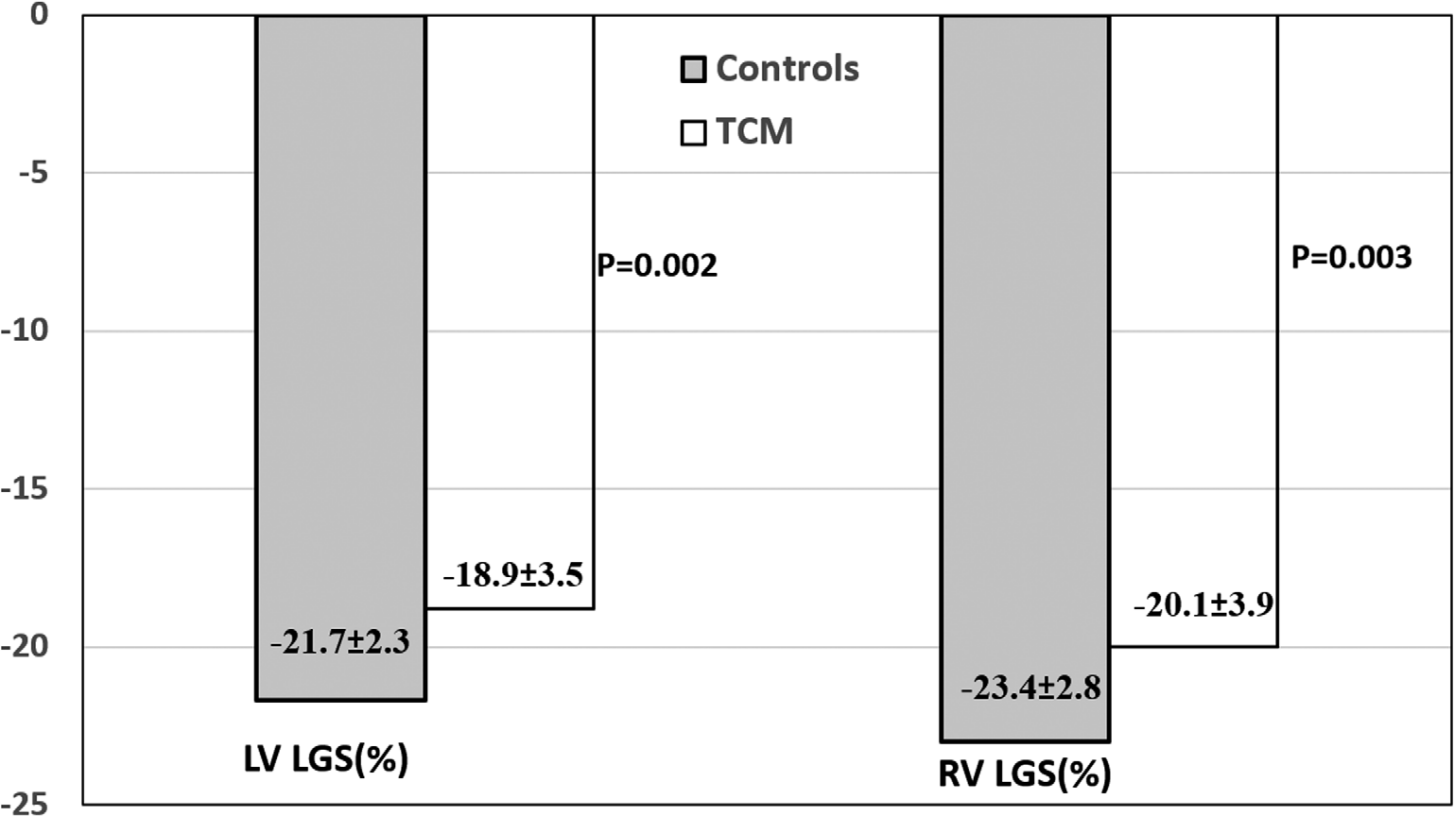
Comparison 2‐dimentional strain values between patients with prior Takotusubo cardiomyopathy (TCM) and controls. LGS, longitudinal global strain; LV, left ventricle; RV, right ventricle

2D strain analysis demonstrated significantly impaired global average LV longitudinal strain (LGS) (−18.9 ± 3.5 vs. −21.7 ± 2.3, p = .002), and lower early diastolic (1.1 ± 0.2 vs. 1.4 ± 1.5 1/s, p = .004) and early systolic (−0.9 ± −0.3 vs. −1.1 ± −1.0 1/s, p = .008) SR compared to the controls at the enrollment. However, no differences were found in circumferential strain at the papillary muscle level (−19.4 ± 4.2 vs. −18.5 ± 9.9, p = .7). The apical level (−22.2 ± 6.9 vs. −23.8 ± 9.3, p = .5) tended to be lower but did not reach statistical significance. Significantly lower LGS of the RV free wall was seen in TCM patients (*n* = 26) compared to controls (−20.1 ± 3.9 vs. −23.4 ± 2.8, p = .003 (Figure 1). Only five patients had biventricular dysfunction at presentation, exhibiting lower RV strain at follow‐up compared with patients with isolated LV dysfunction at index event (−16.4 ± 1.5 vs. −20.9 ± 4.0, p < .01).

The vast majority of patients were in functional class I (92.9%), and three patients were in class II (7.1%). The 6 month stress echocardiography results revealed normal contractile reserve in all patients. No difference in HRR was observed between TCM patients and healthy controls (39.0 ± 0.9 vs. 39.3 ± 16.7 bpm, p = .88).

### 3.3 EPCs, VEGF, and VEGF‐R circulating levels

**FIGURE 2:**
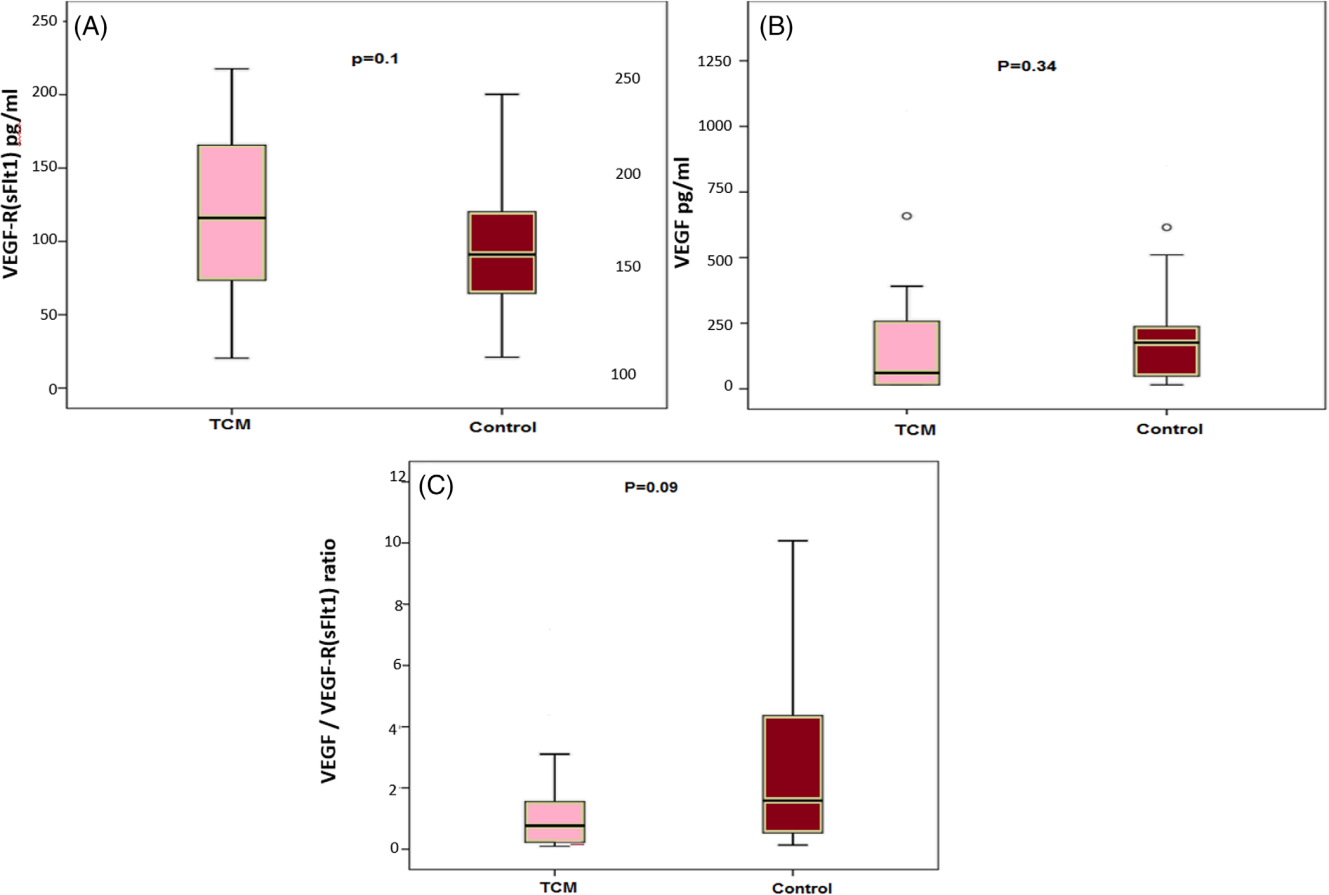
Comparison of VEGF‐R, VEGF and VEGF/VEGF‐R between the TCM patients and controls. Box plots of VEGF‐R (3A), VEGF (3B) and VEGF/VEGF‐R (3C) concentration. The band at the middle of each box plot represents the median, and the bottom and top of the boxes represents the 25th, 75th percentiles. VEGF, vascular endothelial growth factor; VEGF‐R, vascular endothelial growth factor receptor

There was a clear trend toward higher numbers of circulating CD34+ cells in recovered TCM women compared with controls (0.65 [0.32–0.83] vs. 0.03 [0.13–0.43], p = .05), but no significant differences in CD34+/KDR+ numbers in post‐TCM patients when compared with the control group (p = .8) (Figure 1, supplementary file). Higher concentrations of VEGF‐R and lower VEGF/VEGF‐R ratios were found in patients with prior TCM with inadequate increase in VEGF (Figure 2.). Moreover, we found correlation between VEGF‐R/VEGF ratio and average LV longitudinal strain (r = 0.40, p = .014), which may suggest a potential effect of angiogenic markers on residual LV remodeling.

### 3.4 Outcomes at long–term follow‐up

All post‐TCM patients were followed at our heart failure clinic (clinical and echocardiography) every 6 months after their acute presentation. Mean follow‐up was 6.9 ± 1.6 years (median 5.7, max. 10 years min. 2 years). Fifty‐one percent had a follow‐up of more than 5 years.

No stroke, heart failure, death or TCM recurrence, occurred during the first 30 days after admission. No cardiovascular mortality occurred and only two patients died, one from respiratory failure due to severe COPD and the other due to sepsis. Recurrence of TCM occurred in three women, 1, 4 and 6 years after the index event.

## 4 DISCUSSION

In our study, we demonstrated that patients with prior TCM had residual biventricular cardiac dysfunction, including lower LV early velocities (E′) and higher E/E′ ratio by TDI, and impaired both LV and RV GLS compared with the control group at intermediate follow‐up despite LVEF normalization. In addition, there was a trend of higher VEGF‐R concentration with inappropriate levels of VEGF, suggesting some underlying endothelial dysfunction. In addition, lack of in‐hospital mortality and reasonable long‐term outcome of TCM patients were observed.

### 4.1 Residual cardiac impairment

Available data suggest that LVEF assessment by standard echocardiography may not be sensitive enough to mirror complete recovery of LV function. TDI and 2D tracking imaging more accurately assesses both LV and RV function. The 2DS technique for quantification of myocardial strain, provides data on longitudinal and circumferential myocardial function and rotation.^23^ These deformation indices were reported to be sensitive indicators of subtle changes in LV function.^24^, ^25^

In our study we found that patients with prior TCM had significantly lower TDI parameters representing both diastolic and systolic LV dysfunction, including higher LV filling pressures, compared with controls. Analyzing the deformation indexes by 2DS, residual abnormalities in these patients included impaired LV longitudinal strain and SR, but no significant decrease in circumferential strain were observed compared with controls. Notably, all these residual changes occurred in patients with normalized LV function after the index event. It is also important to note that we for the first time, report on an altered RV deformation concomitant with residual LV dysfunction in patients with prior TCM.

A few studies investigated post‐TCM LV function using comprehensive echocardiography and/or cardiac MRI with conflicting results. In contrast to our findings, Mansencal et al. observed no significant differences between the TCM and a group of healthy volunteers in mean values of segmental systolic peak velocity, strain and SR, at early one‐month follow‐up.^26^ Meimoun et al. evaluated LV twist mechanics in 17 patients with TCM during the acute phase and during early follow‐up. They found complete recovery of previously impaired LV twist mechanics at one‐month post the acute event.^27^

Contrary to these reports, supporting our findings, Neil et al. showed that in 36 TCM patients, recovery of the LVEF was obtained within 3 months and GLS, SR, and apical twist improved significantly. However, they found that GLS remained reduced compared to controls even at the three‐month follow‐up.^28^

Subsequently, this group compared 52 patients with TCM to 44 healthy control subjects using MRI.^18^ At 4‐months after the TCM acute episode, there were still significant GLS impairment and apical circumferential strain (p˂0.01), but with no differences in TDI parameters compared with healthy controls despite LVEF normalization.

Nowak et al. investigated LV function recovery at 6 months after discharge among TCM patients.^17^ These investigators described only residual abnormalities in LV apical rotation, significantly lower mean systolic apical rotation, mean peak early diastolic rotation rate compared to the healthy controls, and no difference in diastolic parameters on TDI and GLS. Recently, Scally et al. compared 37 patients with prior (>12‐month) TCM after 20 (range 13–39) months to 37 age‐sex and comorbidity‐matched controls.^19^ This study showed that despite normal LVEF and serum biomarkers (BNP), patients with prior TCM at a median time of 20 months from the index event had impaired cardiac deformation indices by means of both reduced apical circumferential strain.

RV involvement has been described in 24% of patients at acute presentation of TCM, usually in patients in whom the LVEF was significantly lower.^29^ However, serious complications such as cardiogenic shock and pulmonary edema were not more frequent in these patients..^30^ Significant RV dysfunction was found in eight of the 25 TCM patients and was associated with severe HF or cardiopulmonary resuscitation. Seven patients had echocardiography at follow‐up (7 ± 6.3 months) and showed visually assessed improvements of their LV and RV functions..^31^

There is scarce data on RV deformation indices after an acute TCM event.

Heggemann et al. followed 28 TCM patients, half with biventricular involvement. The authors reported on impaired longitudinal RV strain at the index event in patients with RV involvement which improved upon follow‐up.^32^ In our study, lower RV strain was obtained at follow‐up in patients with concomitant RV dysfunction at index event compared to patients with isolated LV dysfunction. Morover, significantly lower RV LGS was found in TCM patients compared with controls suggesting that RV involvement may be underestimated during the acute phase, because the visual evaluation only that is often used in clinical practice is not sensitive enough for RV assessment.

### 4.2 Endothelial dysfunction in prior TCM patients

The precise mechanisms leading to TCM remain unclear. An endothelial imbalance that may interplay between emotional or physical stress and myocardial dysfunction in TCM may be involved in the pathophysiology of this syndrome.^5^, ^6^, ^33^ This hypothesis was evaluated by Naegele M et al.^5^ Endothelial dysfunction was measured on the brachial artery as flow‐mediated dilatation in 22 TCM patients and compared to 21 matched controls. Significantly impaired endothelial function was seen in TCM patients. These findings suggest that women with TCM may have underlying ED causing not only alterations in peripheral vascular reactivity but may also predispose coronary artery vascular dysfunction. Since ED is suggested to represent an important link between stress and transient myocardial dysfunction, we evaluated surrogate biomarkers of endothelial function in our patient population.

EPCs are peripherally increased in response to any vascular injury by mobilization of cytokines such as VEGF and others, and are potential biomarkers for cardiomyopathies.^34^ EPCs elevation was described in congestive heart failure and the severity was associated with elevation in the early phase and depression in the advanced phases of the disease.^10^, ^11^ In our recently published study on women who recovered from peripartum cardiomyopathy, we found significantly higher sFlt1 (VEGF‐R) levels with a clear trend toward lower circulating CD34+/KDR+ levels compared to healthy controls, suggesting an endothelial imbalance.^9^ In the current study, we found a trend for higher numbers of circulating CD34+ cells. However, CD34+/KDR+ numbers in recovered TCM were similar to the controls. Additionally, a higher concentration of VEGF‐R and decreased VEGF‐R/VEGF ratio with inadequate levels of VEGF were evident in post‐TCM patients compared to the control group. VEGF‐R is a potent decoy receptor that acts as a VEGF inhibitor which plays an important role in promoting vasculogenesis. Increased levels of VEGF‐R produced by pericytes from different tissues with inappropriate production of VEGF were described in atherosclerosis, chronic kidney disease, diabetes mellitus, and patients with a history of preeclampsia and peripartum cardiomyopathy,^9^, ^35^, ^36^ and was proposed as an important factor contributing to ED in this population. There are no previous studies looking at endothelial function biomarkers in post‐TCM patients. Based on our findings, we propose that the lack of compensatory enhanced production of VEGF as a response to elevated serum inhibitor VEGF‐R may lead to angiogenic imbalances and consequent predisposition to stressors resulting in a reversible stress induced cardiomyopathy. It should be noted that endothelial dysfunction was reflected only by endothelial function biomarkers, and was not supported by the presence of autonomic function impairment evaluated by HRR during stress echocardiography.

### 4.3 Clinical assessment and outcomes

The prognosis and recurrence rate has been recently shown to be worse than initially thought. Recently, a high in‐hospital mortality has been reported up to 5%.^15^, ^36^, ^37^ A number of reports describe a relatively high long‐term mortality and recurrence rates. Pelicca et al. found an annual rate of 3.5% total mortality (95% CI: 2.6%–4.5%) and 1.0% annual rate of recurrence (95% CI: 0.7%–1.3%) in a large systematic review that included 4679 patients with TCM.^3^ Citro et al. reported 2.8% recurrence, 7.4% mortality, and 4.7% cardiac death at a median of 26.5 months follow‐up among 326 patients with TCM enrolled in the Takotsubo Italian Network.^38^ In our study, favorable outcomes of patients with TCM were observed with no cases of in‐hospital mortality and low rates of acute complications that can be probably explained by early admission and treatment (<8 h from symptoms occurrence). In line with previous studies, recurrence occurred in 7% of our patients.

Although normal LVEF along with persistent symptoms and reduced exercise capacity have been reported in patients recovered from TCM,^19^, ^28^ in our study, patients with prior TCM were free from heart failure symptoms and showed normal LV contractile reserve at 6 months. Medications were used in a minority of patients, mostly for hypertension treatment.

## 5 CONCLUSION

Residual alteration of cardiac function by tissue Doppler and speckle‐ tracking imaging were found in patients recovered from TCM despite normalized LVEF. Inappropriate production of VEGF and VEGF‐R were observed even after LV recovery in patients with Takotsubo cardiomyopathy, suggesting a possible underlying endothelial dysfunction in these patients. Future larger studies are needed to evaluate residual endothelial dysfunction in this patient population.

## 6 LIMITATIONS

The main limitation of our study is a relatively small number of patients including females only. Therefore, the differences in endothelial function biomarkers did not reach statistical significance and showed only a trend compared with controls. Despite it, all underwent an extensive workup and were followed more than 5 years and we were able to report complete recurrence and mortality data.

## CONFLICT OF INTEREST

The authors have no conflicts of interest to disclosure.

## Supporting information


**Figure S1** Comparison of EPC's (CD + 34 and CD + 34/KDR) between the TCM patients and controls
Box plots of CD34+ (2A) and CD34+/KDR+ (2B) concentration. The band at the middle of each box plot represents the median, and the bottom and top of the boxes represents the 25th, 75th percentiles.

## Data Availability

The data that support the findings of this study are available from the corresponding author upon reasonable request.
