# Supplementary figures and images for: Residual alterations of cardiac and endothelial function in patients who recovered from Takotsubo cardiomyopathy
Source: Clin Cardiol. 2021 May 6;44(6):797–804. doi: 10.1002/clc.23604 (PMC8207966; doi:10.1002/clc.23604)

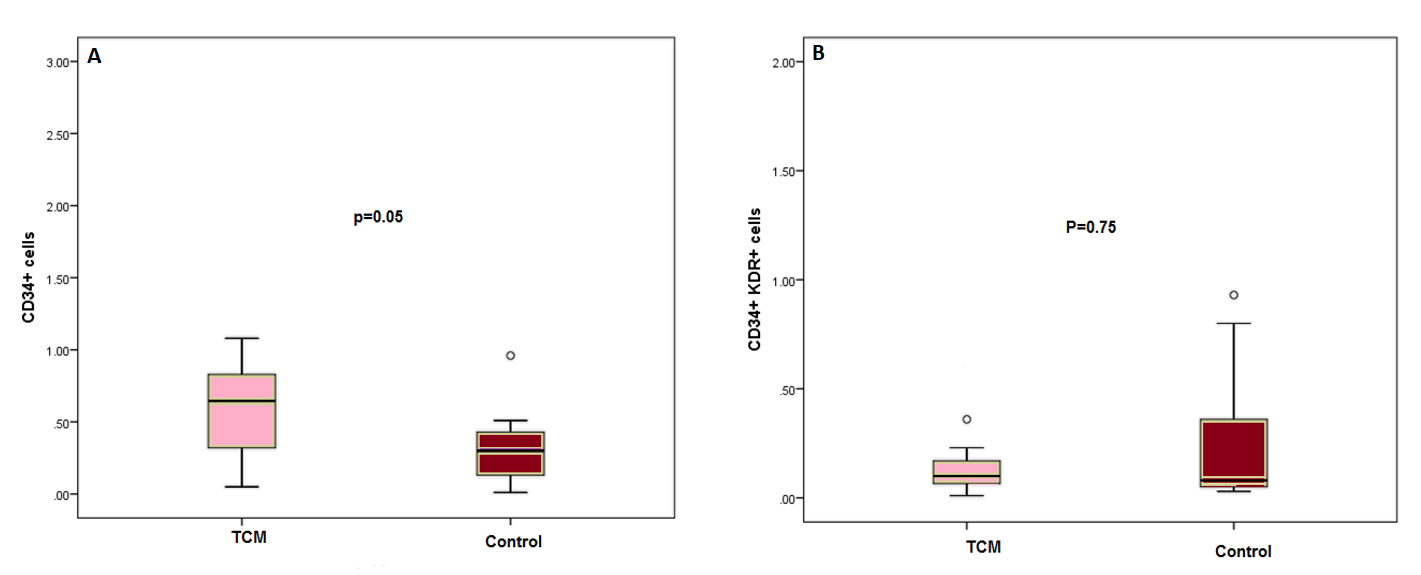

Supplement: Supplementary file 1 — Figure S1 Comparison of EPC's (CD + 34 and CD + 34/KDR) between the TCM patients and controls Box plots of CD34+ (2A) and CD34+/KDR+ (2B) concentration. The band at the middle of each box plot represents the median, and the bottom and top of the boxes represents the 25th, 75th percentiles. [file CLC-44-797-s001.tif]
